# Rationalism in Impression Work

**Published:** 1920-01

**Authors:** F. W. Frahm

**Affiliations:** Los Angeles, Cal.


### RATIONALISM IN IMPRESSION WORK

BY F. W. FRAHM, D.D.S., LOS ANGELES, CAL.

Allow me to preface this essay with the statement that the questionable reliability of some brands of impression material, the prevailing ignorance and carelessness concerning their manipulation, use and application are, no doubt, factors that militate against the success of many denture operations.

Our literature offers many solutions for the problems of impression taking; many different technics are offered, each promising the only solution or specific for our difficulties, disclaiming all weaknesses and promising features that can not be maintained. It may be that these deficiencies are not due to any one cause in particular, and therefore can not be charged directly to the method, but to some misunderstanding or misapplication of it. Neither could the difficulties be laid at the door of the technician who is using the method to the best of his ability and understanding, for the one real factor, the patient, remains the same. Frequently, our apparent successes prove a miserable failure on account of this factor. It we were dealing with unchanging conditions and stable factors, we might develop one technic to successfully care for all cases. Be that as it may, we must recognize the fact that one method will not always, and in all cases, insure the uniform results that we really expected.

When we meet with unfavorable results, or transitory success, would it be unreasonable to charge it to an unfortunate selection of a method for a given case?

Should we always charge unsuccessful results to our lack of understanding and inability to carry out the intricate technic of a difficult method, or may we not enter the plea of over-enthusiasm of the originator or sponsor of the method?

Would it not be unscientific for us to use empirical methods and expect to get uniform results in all cases, having a full knowledge of their clinical requirements?

Would a scientific man disregard rational methods in order that he might carry out some pet ideas which are more or less of an empirical character, and then blame the factor or the materials?

The writer is of the opinion that empiricism has a place in some of our methods. He is also conscious of the fact that some empirical methods will some day become rational, but why should we at any time discard a rational method, and turn to some which are undergoing constant changes, or may even be discarded?

Dr. J. W. Greene, who without doubt is father of the modeling compound impression technic, had, through all the years of his work, a consciousness of an element of deception in it, that favored his technic and gave him his apparent success. This element of deception soon appeared after the denture had been worn by the patient for a month or so. It does not manifest itself at once, but comes in so gradually that the patient does not understand it, and, some times even the operator does not understand this deception.

We may not agree regarding the relative merits of the three or four impression methods now in common use. But, I think you will agree with me, that we should try to establish fundamental standards that can be recognized as basic and broad enough for several men with different technics to build upon.

Dr. Buckley recently said: "It is wrong for any man to claim that there is only one method or means of accomplishing definite results, and that that method is the one he champions, or has originated." Such a position is wrong and untenable. It would discourage many men, who would otherwise become more proficient and of great service to a larger per cent. of the human race now suffering from the lack of real denture service.

It is wrong and unscientific to ask any man, or set of men, to master a specific lengthy technic in impression taking, to the exclusion of all others; or else advise these denture patients to go elsewhere. Such a course might not be convenient for the patient; neither would it be just to either patient or operator. The general practitioner can not be expected to specialize in everything his profession may offer.

Some men may choose to follow some special branch of practice, and develop a high standard of technic for this especial field, but, the greater per cent. of our men will have to follow a general practice with its limited opportunities for development and practice of selected technics. Such men must have a workable technic that will offer the largest returns for a given amount of time. For their benefit we should develop several simplified methods that will bring the largest possible degree of success.

When certain general requirements have been recognized, we should develop as many proven methods as we can, to meet these requirements in a scientific manner. Then those men who would practice prosthesis could select the method best adapted to their degree of ability. They will then have the feeling that they are doing something worth while, and of service to their patients.

At this time the student in prosthesis finds himself confronted with four methods of procedure for impression taking of edentulous mouths. The technic in each method is radically different from that of the others, but the results of these methods (with one exception) are very similar. This must be our unbiased conclusion, if we have a mind open to conviction and are willing to receive the truth, when it is faithfully presented and clinically demonstrated.

Since good results are to be had from any one of the several methods now in use, there must be some underlying fundamental principles common to all these methods. The factors must be the same and must harmonize with the physical forces involved. These physical forces are the same for every case, but their influence may be somewhat modified by factors, which are perhaps carelessly considered or readily overlooked.

Let us consider two forces that aid us in both the maxillary and mandibular arch of each case; namely, adhesion and cohesion. Then the force of gravity militates against the maxillary case and favors the mandibular case. Also atmospheric pressure of 14.7 pounds per square inch at sea level. This force must be considered of value for retention of the denture for either arch.

The four factors that enable us to avail ourselves of these forces are: relief of muscle strain, peripheral bearing, areal contact, and extension for retention. Any technical procedure that will enable us to use these factors, in compelling the retaining forces to work for us, is scientific and worthy of our consideration, regardless of the nature of the materials we may employ.

Any technical procedure that is simply elaborate is, to that extent, undesirable and should be simplified; or it should give way to a simpler technic, providing that such simplicity does not reduce the efficiency of the future denture.

Adhesion is the force that attracts or holds unlike molecules one to another and whose planes are posed at nearly right angles to the displacing force. Example:

One polished plane resting upon another, thus excluding the air.

Cohesion is the force that attracts like molecules one to another, whose planes are posed at nearly a right angle to the displacing force. Example: Two pieces of plate glass resting on each other. The combination of these two forces with the presence of moisture are of value to us for retention. Adhesion of denture to the tissues and saliva, and the cohesion of one molecule of saliva to another.

For mandibular cases we may expect these two forces, with the addition of gravity and atmospheric pressure, to aid us in retaining the case; while in the maxillary cases we take advantage of atmospheric pressure, cohesion and adhesion. In addition to these we can in selected cases utilize some muscles for retention.

Let us consider the factors that enable us to use these forces to the best possible advantage.

### MUSCLE TRIMMING

J. W. Green, who without doubt was the first man to speak of muscle trimming, or "very-edge" impression, and who developed a workable technic to enable us to obtain a tested impression, said: "Muscle trimming is *quick work.*" And, by the way, you can, by this "very-edging" process, trim the impression (hence the plate) more than ten times quicker than you could file or trim a plate, not considering the greater accuracy in favor of muscle trimming. No language can overpraise the proper conformation of the rim of a plate to the yielding tissues.

This may be done by several methods:

1. *Manual methods.* Not many years ago this trimming was done entirely after vulcanization. Some men are doing it this way even at the present time. The method is faulty, tedious and costly to both patient and practitioner, and it is too unreliable for a scientist to use.

Then there is the method of trimming of the impression with repeated cutting of a knife in order that it may not impinge upon the muscles. This method has been followed by a few men for some time and is at the present endorsed by a large number of men. The method has merit and can be used by more men of limited technical ability than some of the other methods, but there will always remain the element of uncertainty regarding the correctness of the amount of trimming that should be done.

2. *Automatic methods.* These methods differ from the others in that the tissues themselves cut their seat or clearance in a plastic material that will harden at normal mouth temperature.

The first, or wax, is perhaps the older of the two methods. It is too uncertain to receive a great deal of attention; however, in the hands of a careful operator it will render good service. After the correct tray has been selected, its periphery is then banked with yellow beeswax. This is then softened and placed in position in the mouth with the hope that muscle activity will cut through and clear itself, thus allowing a certain amount of compression of the remaining tissues. This operation may be repeated several times to insure clearance for the muscles that would otherwise force the denture from its seat. This modified or muscle-trimmed tray is to form the base of a plaster impression.

The other method in this class is known as the Greene-Supplee method. A carefully selected tray is treated with a layer of modeling compound, which after the first or basic application should be of a nearly uniform thickness. This is followed by a more or less elaborate technic to obtain the desired length and flanges, which, by repeated heating or softening of the borders, will allow the lip, tongue frenum and muscles to cut down the periphery and establish, by various activities, the border outline of the denture. The patient, who has been previously coached regarding the muscular gymnastics that he is to perform, will by these methods, and with the aid of the operator, adapt the periphery of the impression to such a degree that it will require a special effort on his part to dislodge it.

The second fundamental feature that must be had in a successful impression is areal contact. Without uniform areal contact, we can never expect to have any permanent success. The establishment of a partial vacuum for a seat to a denture is a delusion and not satisfactory. It will soon vanish, and in its place there will be found as much abnormal soft tissue as such a space will allow. When this has taken place we will have areal contact, but it is now established on a yielding abnormal foundation. A denture that is built upon such a foundation will prove a double disappointment to the patient and operator. Therefore, great care must be exercised to obtain real areal contact.

There are only two methods whereby we can get real areal contact; namely, the Green-Supplee modeling compound method and the Hall compound-plaster method. Of the two methods, I think the Hall method will insure success for the largest number of cases in the practice of the ordinary dentist, because the small amount of plaster that is necessary will seek out every area alike and reproduce its contour. By the use of the small hole in the center of the vault, we will maintain peripheral bearing without logging the palatal portion of our impression with compressed plaster.

Any plaster impression technic that will permit a large amount of plaster in the palatal portion of the tray will not give a true likeness of that part owing to the sagging nature of the material. Plaster, like modeling compound, must be confined in as even and thin a layer as possible in order that we may prevent the volume change that usually accompanies careless manipulation.

3. *Peripheral bearing.* In dealing with a foundation that is more or less yielding in character, it becomes necessary that we obtain peripheral bearing—but without impinging upon the muscles actively engaged in mastication and degluitition, if we expect to obtain satisfactory results in denture work.

To obtain peripheral bearing, we must have a material that will be plastic enough to yield to a certain degree under the pressure of muscles and their attachments, yet rigid enough to be carried to those parts that will enable us to take advantage of all the retention a given case may offer. Without doubt, this material is modeling compound. It is really unnecessary to state that only the modeling compounds especially prepared for this purpose should be used. Beeswax offers some possibilities along this line, but has no added advantages over modeling compound. In the hands of Dr. George H. Wilson, beeswax seems to be a very desirable material, and no doubt he obtains very good results with it. However, I prefer modeling compound.

4. *Extension for retention.* This is also a necessity. It has been emphasized by men of each method and it plays an important part in our successful denture. But, we can all remember satisfactory cases that never were extended. The reason they were successful is because the operator obtained *a real contact.* The two materials that enable us to obtain retention by extension, are modeling compound and beeswax. We will allow compound to have the first place, compound and wax the second place, and beeswax the last place.

5. *Tissue compression.* We must not ignore tissue compression when we encounter an area of mucous tissue, that is, of varying density. These differences must be accounted for at several stages during the course of our denture operation. We can not hope to carry the equalization made in the impression on through to the finished denture without some loss. However, we can embody this tissue compression in the cast and to a large extent in the denture. But there should be a readjustment made before the denture is completed. These impressions must be made with a material other than plaster, namely, modeling compound or beeswax. The question of when we should equalize under biting stress is a debatable one. It may be cared for at two stages of the denture operation.

### POST DAMMING

Dr. Green in commenting on this part of the technic says: "This is really the most important point for adjustment in the whole impression." He then outlines four technical methods for completing the muscle-trimmed impression.

### CONCLUSION

In conclusion I shall try to outline what I consider a rational course in impression work for an edentulous case. A thorough examination of the oral cavity and related structures, with a careful charting of everything that may have a bearing on the construction and use of the appliance, should first be made.

A careful consideration of the forces and factors that will prove advantageous in the construction of the denture should be made; also the forces and factors that will militate against our success.

Having gained a knowledge of the tissues upon which we are to construct our denture and the forces for and against us, we must decide on the technic best adapted to our ability and to the case in hand. Then follows this technic. Use the materials that have proven best suited to such a technic. If you think the Greene-Supplee method is best adapted to your needs and understand it, use it and follow its technic to the letter. Do not at any time follow a slip-shod course and then blame the method or materials for your failure. If in your judgment the case can be best handled with the Greene-plaster method, or Dr. Hall's modification of it, use it and do not depart one step from the fundamental technical points involved. Such departure may mean the failure of the case. If you have mastered the Wilson method and can handle it successfully, use it if it is indicated for that case; but remember that you will have to contend with a volumetric change that occurs in a large, uneven body of plaster. If you will take advantage of the controls in this method, you may expect a reasonable degree of success.

Take advantage of all the forces at your command; compel the factors that enable you to control these forces to work for you and insure a successful denture operation.—*Pacific Dental Gazette.*

